# 2-{[4-(1,3-Benzothia­zol-2-yl)phen­yl](meth­yl)amino}acetic acid

**DOI:** 10.1107/S1600536809041452

**Published:** 2009-10-17

**Authors:** Yong Zhang, Bi-lin Zhao

**Affiliations:** a School of Chemical and Materials Engineering, Huangshi Institute of Technology, Huangshi 435003, People’s Republic of China

## Abstract

In the title compound, C_16_H_14_N_2_O_2_S, the dihedral angle between the benzothia­zole ring system and benzene ring is 3.11 (2)°. In the crystal structure, inter­molecular O—H⋯N hydrogen bonds link mol­ecules into chains along [100] and these chains are, in turn, linked into a three-dimensional network *via* weak inter­molecular C—H⋯O hydrogen bonds.

## Related literature

In an effort to develop in vivo β-sheet imaging probes, many derivatives of thio­flavin T, a water-soluble fluorescent dye, have been synthesized and evaluated, see: Kung *et al.* (2001 ▶); Qu *et al.* (2007 ▶). For the synthetic procedure, see: Stephenson *et al.*, 2007 ▶.
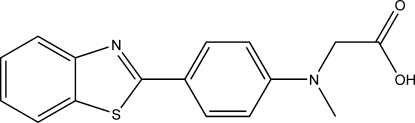


## Experimental

### 

#### Crystal data

C_16_H_14_N_2_O_2_S
*M* *_r_* = 298.35
Orthorhombic, 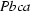

*a* = 11.9516 (10) Å
*b* = 9.4390 (8) Å
*c* = 25.418 (2) Å
*V* = 2867.5 (4) Å^3^
*Z* = 8
Mo *K*α radiation
μ = 0.23 mm^−1^
*T* = 298 K
0.16 × 0.15 × 0.12 mm

#### Data collection

Bruker SMART CCD diffractometer
Absorption correction: multi-scan (*SADABS*; Sheldrick, 1996 ▶) *T*_min_ = 0.964, *T*_max_ = 0.973
12100 measured reflections
3234 independent reflections
2156 reflections with *I* > 2σ(*I*)
*R*_int_ = 0.057

#### Refinement

*R*[*F* ^2^ > 2σ(*F* ^2^)] = 0.059
*wR*(*F* ^2^) = 0.134
*S* = 1.06
3234 reflections
194 parameters
H atoms treated by a mixture of independent and constrained refinement
Δρ_max_ = 0.26 e Å^−3^
Δρ_min_ = −0.18 e Å^−3^

### 

Data collection: *SMART* (Bruker, 2007 ▶); cell refinement: *SAINT-Plus* (Bruker, 2007 ▶); data reduction: *SAINT-Plus*; program(s) used to solve structure: *SHELXS97* (Sheldrick, 2008 ▶); program(s) used to refine structure: *SHELXL97* (Sheldrick, 2008 ▶); molecular graphics: *PLATON* (Spek, 2009 ▶); software used to prepare material for publication: *SHELXTL* (Sheldrick, 2008 ▶).

## Supplementary Material

Crystal structure: contains datablocks global, I. DOI: 10.1107/S1600536809041452/lh2913sup1.cif

Structure factors: contains datablocks I. DOI: 10.1107/S1600536809041452/lh2913Isup2.hkl

Additional supplementary materials:  crystallographic information; 3D view; checkCIF report

## Figures and Tables

**Table 1:** Hydrogen-bond geometry (Å, °)

*D*—H⋯*A*	*D*—H	H⋯*A*	*D*⋯*A*	*D*—H⋯*A*
O1—H1⋯N1^i^	1.00 (3)	1.71 (3)	2.695 (3)	167 (2)
C4—H4⋯O2^ii^	0.93	2.50	3.368 (3)	156
C14—H14*A*⋯O2^iii^	0.97	2.47	3.242 (3)	137

Symmetry codes: (i) 
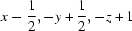
; (ii) 
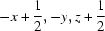
; (iii) 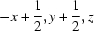.

**Fig. 1.**
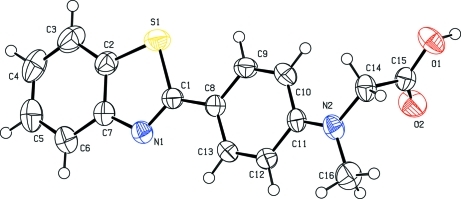
The molecular structure of (I), with displacement ellipsoids drawn at the 50% probability level.

**Fig. 2.**
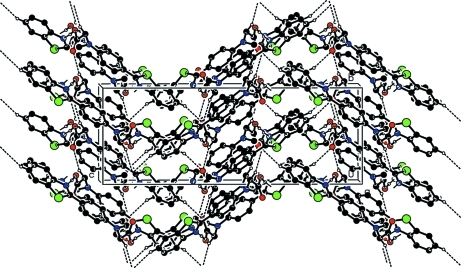
Part of the crystal structure of (I) showing hydrogen bonds as dashed lines. Only H atoms involved in hydrogen bonds are shown.

## supplementary crystallographic information

### Comment

Thioflavin T (ThT) as a water-soluble fluorescence dye has been drawing great attention due to its ability to label amyloid fibrils. In an effort to develop in vivo beta-sheet imaging probes, many derivatives of thioflavin T compounds have been synthesized and evaluated (e.g. Kung *et al.*, 2001; Qu *et al.*, 2007). In this context, we have synthesized the title compound and report its crystal structure herein.

In the molecular structure (Fig. 1), the dihedral angle between the benzothiazole unit and benzene ring is 3.11 (2), and the conformation of the substituted methylamino group is defined by the C16—N2—C14—C15 torsion angle of 86.8 (3)°. All bond lengths and bond angles are as expected. In the crystal structure, intermolecular O-H···N hydrogen bonds link molecules into one-dimensional chains and these chains, are in turn linked into a three-dimensional network via weak intermolecular C—H···O hydrogen bonds (Fig. 2).

### Experimental

Compound (I) was synthesized according to the method described by Stephenson *et al.* (2007). Yellow single crystals suitable for an X-ray diffraction study were obtained by slow evaporation of an methanol solution of the title compound.

### Refinement

All H atoms were placed in idealized positions [C–H=0.96 Å (methyl), 0.97Å (methylene) and 0.93 Å (aromatic)] and included in the refinement in the riding-model approximation, with *U*_iso_(H)= 1.5*U*_eq_(methyl C) and 1.2*U*_eq_(methylene and aromatic C). The H atom bonded to the carboxyl group O atom was found from the difference map. The O—H distance was refined freely with *U*_iso_(H)= 1.5*U*_eq_(O)

### Crystal data

**Table:**

C_16_H_14_N_2_O_2_S	*F*(000) = 1248
*M**_r_* = 298.35	*D*_x_ = 1.382 Mg m^−^^3^
Orthorhombic, *P**b**c**a*	Mo *K*α radiation, λ = 0.71073 Å
Hall symbol: -P 2ac 2ab	Cell parameters from 1634 reflections
*a* = 11.9516 (10) Å	θ = 2.3–22.4°
*b* = 9.4390 (8) Å	µ = 0.23 mm^−^^1^
*c* = 25.418 (2) Å	*T* = 298 K
*V* = 2867.5 (4) Å^3^	Block, colorless
*Z* = 8	0.16 × 0.15 × 0.12 mm

### Data collection

**Table:**

Bruker SMART CCD diffractometer	3234 independent reflections
Radiation source: fine-focus sealed tube	2156 reflections with *I* > 2σ(*I*)
graphite	*R*_int_ = 0.057
φ and ω scans	θ_max_ = 27.5°, θ_min_ = 2.3°
Absorption correction: multi-scan (*SADABS*; Sheldrick, 1996)	*h* = −15→6
*T*_min_ = 0.964, *T*_max_ = 0.973	*k* = −11→12
12100 measured reflections	*l* = −31→31

### Refinement

**Table:**

Refinement on *F*^2^	Primary atom site location: structure-invariant direct methods
Least-squares matrix: full	Secondary atom site location: difference Fourier map
*R*[*F*^2^ > 2σ(*F*^2^)] = 0.059	Hydrogen site location: inferred from neighbouring sites
*wR*(*F*^2^) = 0.134	H atoms treated by a mixture of independent and constrained refinement
*S* = 1.06	*w* = 1/[σ^2^(*F*_o_^2^) + (0.0495*P*)^2^] where *P* = (*F*_o_^2^ + 2*F*_c_^2^)/3
3234 reflections	(Δ/σ)_max_ < 0.001
194 parameters	Δρ_max_ = 0.26 e Å^−^^3^
0 restraints	Δρ_min_ = −0.18 e Å^−^^3^

### Special details

**Table:**

Geometry. All e.s.d.'s (except the e.s.d. in the dihedral angle between two l.s. planes) are estimated using the full covariance matrix. The cell e.s.d.'s are taken into account individually in the estimation of e.s.d.'s in distances, angles and torsion angles; correlations between e.s.d.'s in cell parameters are only used when they are defined by crystal symmetry. An approximate (isotropic) treatment of cell e.s.d.'s is used for estimating e.s.d.'s involving l.s. planes.
Refinement. Refinement of *F*^2^ against ALL reflections. The weighted *R*-factor *wR* and goodness of fit *S* are based on *F*^2^, conventional *R*-factors *R* are based on *F*, with *F* set to zero for negative *F*^2^. The threshold expression of *F*^2^ > σ(*F*^2^) is used only for calculating *R*-factors(gt) *etc*. and is not relevant to the choice of reflections for refinement. *R*-factors based on *F*^2^ are statistically about twice as large as those based on *F*, and *R*- factors based on ALL data will be even larger.

### Fractional atomic coordinates and isotropic or equivalent isotropic displacement parameters (Å^2^)

**Table:**

	*x*	*y*	*z*	*U*_iso_*/*U*_eq_	
C1	0.36303 (18)	0.1030 (2)	0.62901 (9)	0.0352 (6)	
C2	0.3131 (2)	−0.0017 (3)	0.71444 (10)	0.0448 (6)	
C3	0.2757 (3)	−0.0521 (3)	0.76272 (11)	0.0604 (8)	
H3	0.2112	−0.0160	0.7783	0.072*	
C4	0.3367 (3)	−0.1562 (3)	0.78658 (11)	0.0654 (9)	
H4	0.3122	−0.1932	0.8185	0.079*	
C5	0.4342 (3)	−0.2081 (3)	0.76424 (11)	0.0599 (8)	
H5	0.4747	−0.2778	0.7817	0.072*	
C6	0.4720 (2)	−0.1582 (3)	0.71657 (10)	0.0483 (7)	
H6	0.5374	−0.1937	0.7017	0.058*	
C7	0.4106 (2)	−0.0537 (3)	0.69110 (10)	0.0386 (6)	
C8	0.36685 (18)	0.1881 (2)	0.58153 (9)	0.0347 (6)	
C9	0.28533 (19)	0.2883 (3)	0.56974 (10)	0.0408 (6)	
H9	0.2251	0.2993	0.5926	0.049*	
C10	0.2907 (2)	0.3716 (3)	0.52558 (10)	0.0422 (6)	
H10	0.2334	0.4357	0.5188	0.051*	
C11	0.38126 (18)	0.3618 (2)	0.49032 (9)	0.0344 (6)	
C12	0.46109 (19)	0.2571 (3)	0.50147 (9)	0.0409 (6)	
H12	0.5206	0.2438	0.4784	0.049*	
C13	0.45351 (19)	0.1741 (3)	0.54534 (10)	0.0419 (6)	
H13	0.5082	0.1059	0.5512	0.050*	
C14	0.3081 (2)	0.5541 (2)	0.43544 (10)	0.0432 (6)	
H14A	0.3419	0.6291	0.4147	0.052*	
H14B	0.2804	0.5959	0.4678	0.052*	
C15	0.21108 (19)	0.4931 (3)	0.40533 (9)	0.0368 (6)	
C16	0.4788 (2)	0.4267 (3)	0.40869 (11)	0.0550 (8)	
H16A	0.5510	0.4252	0.4252	0.082*	
H16B	0.4765	0.5013	0.3831	0.082*	
H16C	0.4655	0.3374	0.3917	0.082*	
N1	0.43700 (15)	0.0080 (2)	0.64281 (8)	0.0372 (5)	
N2	0.39284 (16)	0.4510 (2)	0.44825 (8)	0.0421 (5)	
O1	0.13080 (15)	0.58713 (18)	0.39831 (8)	0.0520 (5)	
H1	0.065 (2)	0.540 (3)	0.3813 (11)	0.078*	
O2	0.20655 (15)	0.37398 (19)	0.38956 (8)	0.0583 (5)	
S1	0.25427 (6)	0.12637 (8)	0.67437 (3)	0.0549 (3)	

### Atomic displacement parameters (Å^2^)

**Table:**

	*U*^11^	*U*^22^	*U*^33^	*U*^12^	*U*^13^	*U*^23^
C1	0.0333 (12)	0.0360 (14)	0.0363 (14)	−0.0010 (10)	−0.0001 (11)	−0.0032 (11)
C2	0.0506 (15)	0.0454 (15)	0.0384 (15)	0.0003 (13)	0.0038 (12)	0.0027 (13)
C3	0.075 (2)	0.063 (2)	0.0438 (18)	−0.0004 (16)	0.0151 (15)	0.0044 (16)
C4	0.096 (3)	0.064 (2)	0.0361 (17)	−0.0114 (18)	0.0031 (17)	0.0104 (15)
C5	0.075 (2)	0.0537 (19)	0.0507 (19)	−0.0072 (16)	−0.0165 (17)	0.0119 (15)
C6	0.0468 (15)	0.0481 (17)	0.0500 (18)	−0.0014 (12)	−0.0079 (13)	0.0083 (14)
C7	0.0409 (13)	0.0366 (14)	0.0382 (15)	−0.0057 (11)	−0.0041 (12)	0.0019 (12)
C8	0.0352 (12)	0.0349 (13)	0.0339 (14)	−0.0012 (11)	−0.0016 (11)	0.0002 (11)
C9	0.0354 (13)	0.0442 (15)	0.0429 (16)	0.0037 (11)	0.0052 (11)	−0.0003 (13)
C10	0.0396 (13)	0.0380 (14)	0.0490 (17)	0.0071 (11)	−0.0049 (12)	0.0035 (13)
C11	0.0333 (12)	0.0351 (13)	0.0348 (14)	−0.0058 (10)	−0.0085 (10)	−0.0013 (11)
C12	0.0358 (13)	0.0518 (16)	0.0351 (15)	0.0058 (11)	0.0029 (11)	0.0043 (12)
C13	0.0371 (13)	0.0488 (16)	0.0399 (15)	0.0107 (11)	−0.0021 (12)	0.0042 (13)
C14	0.0538 (15)	0.0338 (14)	0.0419 (15)	−0.0024 (12)	−0.0093 (13)	0.0073 (12)
C15	0.0433 (13)	0.0327 (14)	0.0345 (14)	−0.0036 (11)	−0.0002 (11)	0.0057 (11)
C16	0.0535 (16)	0.0594 (19)	0.0520 (18)	−0.0068 (14)	0.0062 (14)	0.0155 (15)
N1	0.0332 (10)	0.0386 (12)	0.0398 (12)	−0.0026 (9)	−0.0011 (9)	0.0040 (10)
N2	0.0408 (11)	0.0411 (12)	0.0444 (13)	0.0004 (10)	−0.0046 (10)	0.0112 (10)
O1	0.0432 (10)	0.0364 (10)	0.0762 (14)	0.0012 (8)	−0.0150 (10)	−0.0006 (9)
O2	0.0598 (12)	0.0422 (12)	0.0727 (14)	0.0036 (9)	−0.0132 (11)	−0.0167 (10)
S1	0.0550 (4)	0.0613 (5)	0.0484 (5)	0.0181 (3)	0.0152 (3)	0.0110 (4)

### Geometric parameters (Å, °)

**Table:**

C1—N1	1.307 (3)	C10—C11	1.408 (3)
C1—C8	1.450 (3)	C10—H10	0.9300
C1—S1	1.752 (2)	C11—N2	1.368 (3)
C2—C3	1.390 (3)	C11—C12	1.403 (3)
C2—C7	1.397 (3)	C12—C13	1.366 (3)
C2—S1	1.730 (3)	C12—H12	0.9300
C3—C4	1.366 (4)	C13—H13	0.9300
C3—H3	0.9300	C14—N2	1.441 (3)
C4—C5	1.386 (4)	C14—C15	1.504 (3)
C4—H4	0.9300	C14—H14A	0.9700
C5—C6	1.377 (4)	C14—H14B	0.9700
C5—H5	0.9300	C15—O2	1.195 (3)
C6—C7	1.390 (3)	C15—O1	1.319 (3)
C6—H6	0.9300	C16—N2	1.456 (3)
C7—N1	1.395 (3)	C16—H16A	0.9600
C8—C9	1.391 (3)	C16—H16B	0.9600
C8—C13	1.392 (3)	C16—H16C	0.9600
C9—C10	1.372 (3)	O1—H1	1.00 (3)
C9—H9	0.9300		
			
N1—C1—C8	125.6 (2)	N2—C11—C12	121.5 (2)
N1—C1—S1	114.31 (18)	N2—C11—C10	122.4 (2)
C8—C1—S1	120.10 (17)	C12—C11—C10	116.1 (2)
C3—C2—C7	121.5 (3)	C13—C12—C11	121.6 (2)
C3—C2—S1	128.9 (2)	C13—C12—H12	119.2
C7—C2—S1	109.53 (18)	C11—C12—H12	119.2
C4—C3—C2	117.8 (3)	C12—C13—C8	122.3 (2)
C4—C3—H3	121.1	C12—C13—H13	118.8
C2—C3—H3	121.1	C8—C13—H13	118.8
C3—C4—C5	121.4 (3)	N2—C14—C15	113.5 (2)
C3—C4—H4	119.3	N2—C14—H14A	108.9
C5—C4—H4	119.3	C15—C14—H14A	108.9
C6—C5—C4	121.1 (3)	N2—C14—H14B	108.9
C6—C5—H5	119.5	C15—C14—H14B	108.9
C4—C5—H5	119.5	H14A—C14—H14B	107.7
C5—C6—C7	118.6 (3)	O2—C15—O1	123.7 (2)
C5—C6—H6	120.7	O2—C15—C14	124.4 (2)
C7—C6—H6	120.7	O1—C15—C14	111.9 (2)
C6—C7—N1	125.9 (2)	N2—C16—H16A	109.5
C6—C7—C2	119.5 (2)	N2—C16—H16B	109.5
N1—C7—C2	114.6 (2)	H16A—C16—H16B	109.5
C9—C8—C13	116.3 (2)	N2—C16—H16C	109.5
C9—C8—C1	122.3 (2)	H16A—C16—H16C	109.5
C13—C8—C1	121.4 (2)	H16B—C16—H16C	109.5
C10—C9—C8	122.2 (2)	C1—N1—C7	111.7 (2)
C10—C9—H9	118.9	C11—N2—C14	121.4 (2)
C8—C9—H9	118.9	C11—N2—C16	121.0 (2)
C9—C10—C11	121.3 (2)	C14—N2—C16	116.5 (2)
C9—C10—H10	119.3	C15—O1—H1	109.4 (16)
C11—C10—H10	119.3	C2—S1—C1	89.89 (12)
			
C7—C2—C3—C4	−0.9 (4)	C10—C11—C12—C13	−2.9 (3)
S1—C2—C3—C4	179.1 (2)	C11—C12—C13—C8	−0.1 (4)
C2—C3—C4—C5	1.6 (5)	C9—C8—C13—C12	2.2 (4)
C3—C4—C5—C6	−1.3 (5)	C1—C8—C13—C12	−177.3 (2)
C4—C5—C6—C7	0.3 (4)	N2—C14—C15—O2	−5.5 (4)
C5—C6—C7—N1	−179.9 (2)	N2—C14—C15—O1	174.4 (2)
C5—C6—C7—C2	0.3 (4)	C8—C1—N1—C7	178.9 (2)
C3—C2—C7—C6	0.0 (4)	S1—C1—N1—C7	0.4 (3)
S1—C2—C7—C6	180.00 (19)	C6—C7—N1—C1	179.8 (2)
C3—C2—C7—N1	−179.8 (2)	C2—C7—N1—C1	−0.4 (3)
S1—C2—C7—N1	0.2 (3)	C12—C11—N2—C14	177.0 (2)
N1—C1—C8—C9	−179.4 (2)	C10—C11—N2—C14	−4.5 (3)
S1—C1—C8—C9	−1.0 (3)	C12—C11—N2—C16	9.8 (3)
N1—C1—C8—C13	0.1 (4)	C10—C11—N2—C16	−171.8 (2)
S1—C1—C8—C13	178.57 (18)	C15—C14—N2—C11	−81.0 (3)
C13—C8—C9—C10	−1.4 (4)	C15—C14—N2—C16	86.8 (3)
C1—C8—C9—C10	178.1 (2)	C3—C2—S1—C1	180.0 (3)
C8—C9—C10—C11	−1.5 (4)	C7—C2—S1—C1	0.00 (19)
C9—C10—C11—N2	−174.9 (2)	N1—C1—S1—C2	−0.23 (19)
C9—C10—C11—C12	3.6 (3)	C8—C1—S1—C2	−178.8 (2)
N2—C11—C12—C13	175.7 (2)		

### Hydrogen-bond geometry (Å, °)

**Table:**

*D*—H···*A*	*D*—H	H···*A*	*D*···*A*	*D*—H···*A*
O1—H1···N1^i^	1.00 (3)	1.71 (3)	2.695 (3)	167 (2)
C4—H4···O2^ii^	0.93	2.50	3.368 (3)	156
C14—H14A···O2^iii^	0.97	2.47	3.242 (3)	137

Symmetry codes: (i) *x*−1/2, −*y*+1/2, −*z*+1; (ii) −*x*+1/2, −*y*, *z*+1/2; (iii) −*x*+1/2, *y*+1/2, *z*.
